# Intramuscular administration of hexachloroplatinate reverses cyanide‐induced metabolic derangements and counteracts severe cyanide poisoning

**DOI:** 10.1096/fba.1024

**Published:** 2018-11-15

**Authors:** Jordan Morningstar, Jangwoen Lee, Tara Hendry‐Hofer, Alyssa Witeof, Tiffany Lyle, Gregg Knipp, Calum A. MacRae, Gerry R. Boss, Randall T. Peterson, Vincent J. Davisson, Robert E. Gerszten, Vikhyat S. Bebarta, Sari Mahon, Matt Brenner, Anjali K. Nath

**Affiliations:** ^1^ Division of Cardiovascular Medicine Beth Israel Deaconess Medical Center Boston Massachusetts; ^2^ Beckman Laser Institute and Department of Medicine University of California Irvine California; ^3^ Deparment of Emergency Medicine University of Colorado School of Medicine Aurora Colorado; ^4^ Department of Comparative Pathology Purdue University West Lafayette Indiana; ^5^ Department of Medicinal Chemistry and Molecular Pharmacology Purdue University West Lafayette Indiana; ^6^ Division of Cardiovascular Medicine Brigham and Women's Hospital Boston Massachusetts; ^7^ Broad Institute Cambridge Massachusetts; ^8^ Department of Medicine University of California San Diego California; ^9^ Department of Pharmacology and Toxicology, College of Pharmacy University of Utah Salt Lake City Utah

**Keywords:** chemical and biological weapons, cisplatin analogues, cyanide toxicity reversal, metabolomics, preclinical large animal model

## Abstract

Cyanide is a highly toxic industrial chemical that is widely used by manufactures. Smoke inhalation during household fires is the most common source of cyanide poisoning while additional risks to civilians include industrial accidents and terrorist attacks. Despite the risks to large numbers of individuals, an antidote capable of administration at scale adequate for a mass casualty, prehospital scenario does not yet exist. Previously, we demonstrated that intravenous cisplatin analogues accelerate recovery from cyanide poisoning in mice and rabbits. Of the dozens of platinum‐based organometallic complexes tested, hexachloroplatinate (HCP) emerged as a promising lead compound, exhibiting strong affinity for cyanide and efficacy across model systems. Here, we show HCP is an antidote to lethal cyanide exposure and is importantly effective when delivered intramuscularly. The pharmacokinetic profile of HCP exhibited bioavailability in the systemic circulation 2.5 minutes post‐treatment and subsequent renal clearance of HCP‐cyanide. HCP restored parameters of cellular physiology including cytochrome c oxidase redox state and TCA cycle metabolism. We next validated these findings in a large animal model (swine). Finally, preclinical safety studies in mice revealed minimal toxicity. Cumulatively, these findings demonstrate that HCP is a promising lead compound for development of an intramuscular injectable cyanide antidote for mass casualty scenarios.

**Abbreviations**
CN: cyanide
DOS: diffuse optical spectroscopy
dpf: days post fertilization
HCP: hexachloroplatinate
TCA: tricarboxylic acid cycle

## 1 INTRODUCTION

Cyanide is a well‐known metabolic poison that is readily absorbed through dermal, bronchial, and digestive routes, rapidly distributes to tissues throughout the body, and causes multi‐organ toxicity.^1^, ^2^ Exposure to the poison in the low milligram range induces symptoms that appear within minutes of exposure.^3^ Cyanide‐induced histotoxic hypoxia underlies the rapid effects of this toxin. By inhibiting cytochrome oxidase, the final complex in the electron transport chain, cyanide blocks the oxygen utilization and hence ATP production.^4^ These downstream effects are particularly deleterious in tissues that have high ATP demands, such as the brain and heart.^5^

Current therapeutic interventions for cyanide poisoning include hydroxocobalamin which acts by reducing the intracellular concentration of cyanide through direct chelation of cyanide anions.^6^, ^7^ Others, sodium nitrite, work by converting hemoglobin to methemoglobin which then avidly binds cyanide, thereby diverting it from binding to cytochrome oxidase.^8^, ^9^ However, nitrite antidotes induce methemoglobinemia which may be deleterious to victims of smoke inhalation, a major accidental cause of cyanide poisoning, because free hemoglobin levels are already lowered due to carbon monoxide.^7^ The third class of FDA approved antidotes, thiosulfates, utilize the endogenous enzyme rhodanese to detoxify cyanide by converting it into thiocyanate; although the reaction occurs too slowly to effectively rescue a poison victim. Though these three classes of antidotes are effective in hospital settings, they require intravenous administration over 15 minutes and often require repeat dosing.^10^ Furthermore, an antidote capable of administration at scale adequate for a mass casualty scenario does not yet exist.^7^ Therefore, the development of a cyanide antidote that can be rapidly deployed, preferably by intramuscular injection, could have a transformative effect on the management of cyanide poisoning.

To that end, we have developed a collaborative pipeline that spans high throughput screening, medicinal chemistry, and mammalian testing, with the overarching goal of countermeasure discovery and preclinical development.^11^ In a high throughput small molecule screen in zebrafish, our group previously demonstrated that cisplatin is a cyanide antidote.^12^ Subsequently, we conducted a structure activity study to identify cisplatin analogues with improved solubility and efficacy. Of the many small molecules tested, we observed concordant protective effects of hexachloroplatinate (HCP) in three vertebrate models of cyanide toxicity.^13^ Further, this compound exhibits an ∼33‐fold improvement in solubility over cisplatin and an ~20‐fold lower reported toxicity in mice than cisplatin (LD_50_ = 133 vs 6.6 mg/kg).^14^ These results provided proof‐of‐concept that cisplatin analogues are effective cyanide antidotes in zebrafish, mice, and rabbits; however, these compounds were administered through intravenous or intraperitoneal administration, limiting their utility in a mass casualty scenario.

The purpose of this study was to establish if HCP is efficacious via intramuscular administration and to undertake initial assessment of the absorption, metabolism, excretion, and toxicity of HCP in mice and rabbits. Additionally, we sought to determine if HCP mitigates cyanide‐induced metabolic derangements. We also test the generalizability of our findings to a large animal, preclinical model. Cumulatively, this study establishes the efficacy, pharmacokinetic profile, and metabolic effects of intramuscular administration of HCP in two clinically relevant mammalian models.

## 2 RESULTS

### 2.1 Intramuscular injection of HCP rescues rabbits from exposure to a lethal dose of cyanide

**Figure 1:**
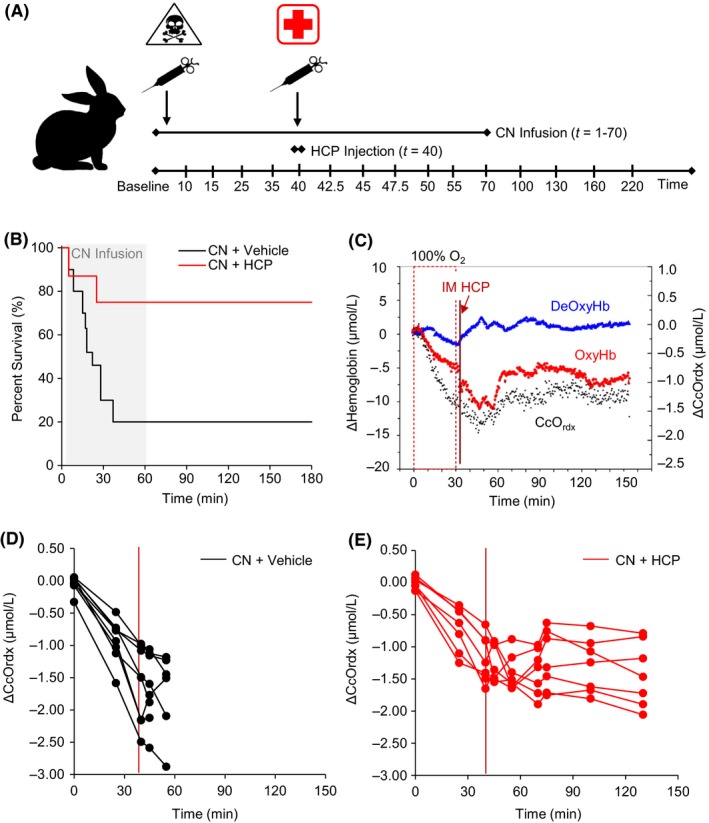
Intramuscular delivery of HCP protects rabbits from a lethal dose of cyanide. A, Overview of experimental procedure in the rabbit cyanide model. B, Kaplan‐Meier plot of HCP (n = 9; red) and saline (n = 11; black)‐treated rabbits exposed to a lethal dose of cyanide. C, Oxygenation hemoglobin (red), deoxygenated hemoglobin (blue), and cytochrome c oxidase redox state (black) in the muscle of a representative rabbit treated with HCP. ΔCytochrome c oxidase redox (µM) in rabbits treated with cyanide and administered D, saline or E, HCP (line denotes when saline or HCP was delivered)

We previously demonstrated that intravenous administration of HCP to rabbits exposed to a sublethal dose of cyanide rapidly reverses cyanide‐induced inhibition of oxygen offloading from hemoglobin and accelerates recovery from cyanide toxicity.^13^ Here, we used an established rabbit protocol in which exposure to cyanide results in death within 40 minutes of cyanide infusion unless an antidote is administered. Lethal cyanide dose was achieved by intravenous administration of 20 mg sodium cyanide (0.33 mg/minutes) until blood pressure dropped below 60 mmHg (~20‐40 minutes), at which time antidote (30 mg/kg HCP IM) or saline was administrated (Figure 1A). Cyanide infusion continued for another 30 minutes. During the experimental sequence, serial blood samples were collected (Figure 1A). This model resulted in lethality for 9 of 11 (81%) saline‐treated rabbits, whereas only two of nine (22%) HCP‐treated rabbits died (*P* = 0.02; Figure 1B). Further these seven rabbits survived the full experimental follow‐up period of 220 minutes demonstrating that HCP is an effective antidote to a lethal dose of cyanide and, importantly establishing that HCP is effective via intramuscular administration.

In this model, cellular cytochrome c oxidase redox state and oxy‐/deoxy‐hemoglobin are monitored using diffuse optical spectroscopy (DOS) from a probe placed on the shaved surface of the right inner thigh muscle (Figure 1C).^6^ Typically, during cyanide poisoning, cellular cytochrome c oxidase redox state steadily decreases over the 40 minute infusion (due to the binding of cyanide anions to iron in cytochrome c oxidase (−0.05 ± 0.02 to −1.43 ± 0.11 µmol/L, *P* = 1E‐10; Figure 1D). By contrast, in animals treated with HCP at t = 40, cytochrome c oxidase redox state plateaus after antidote injection demonstrating HCP halts the deleterious effects of cyanide on cytochrome c oxidase (Figure 1C,E). Concordant protective effects of HCP are observed on the parameters of oxygenated and deoxygenated hemoglobin (Figure 1C; red and blue lines). These results indicate that HCP also restores parameters of cellular physiology.

### 2.2 HCP relieves the TCA cycle blockage induced by cyanide

**Figure 2:**
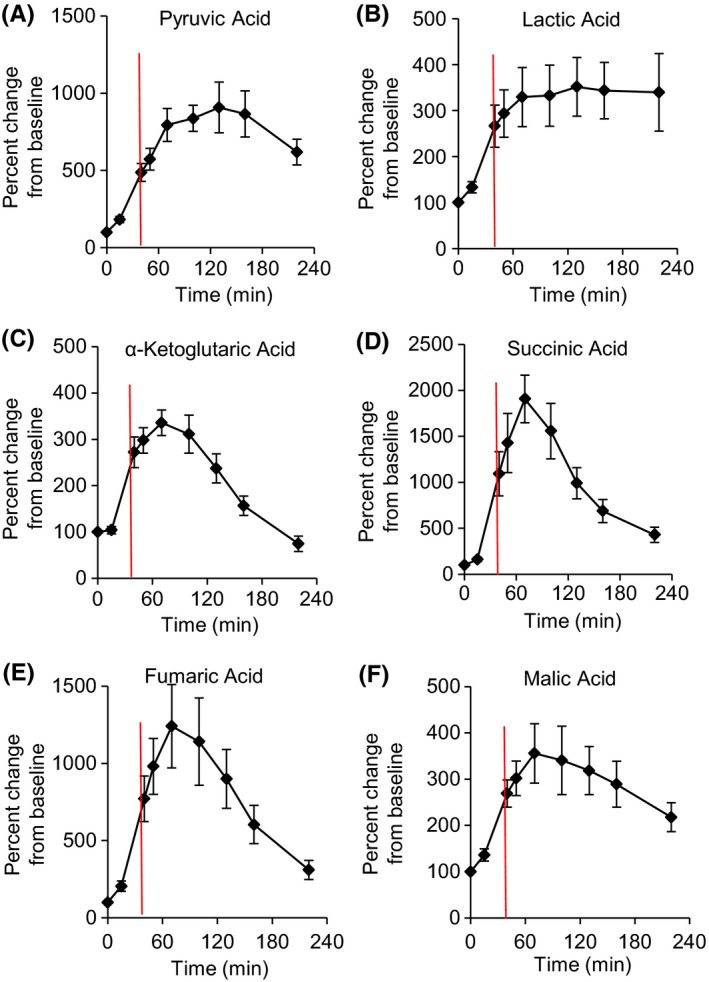
HCP alleviates cyanide‐induced blockage of the TCA cycle. Levels of A, pyruvic acid, B, lactic acid, C, α‐ketoglutaric acid, D, succinic acid, E, fumaric acid, and F, malic acid in rabbits treated with cyanide (at t = 1) and given HCP (at t = 40 minutes, n = 9). Red line denotes when antidote was delivered. Data were normalized to baseline and presented as mean ± SEM

We next used an orthogonal approach to further evaluate the mechanism of antidote efficacy, measuring TCA cycle metabolites by mass spectrometry. As expected, cyanide infusion resulted in significant changes in TCA cycle metabolites due to inhibition of cellular respiration. This led to increased concentrations of TCA cycle metabolites as their consumption slowed down: α‐ketoglutaric acid (+336% ± 27%; *P* = 8E‐05), succinic acid (+1907% ± 259%; *P* = 0.0009), fumaric acid (+1241% ± 270%; *P* = 0.0006), and malic acid (+329% ± 64%; *P* = 5E‐05) (Figure 2A‐F). By contrast, treatment with HCP returned α‐ketoglutaric acid levels to near baseline (+75% ± 17%); excursion of succinic acid (+429% ± 82%), fumaric acid (+310% ± 63%) and malic acid (+218% ± 31%) were also significantly abrogated. Additionally, glycolytic metabolites are increased including pyruvic acid (+794% ± 106%; *P* = 0.001) and lactic acid (+245% ± 64%; *P* = 0.001). These metabolites plateaued after HCP administration. Together these findings indicate that HCP activates TCA cycle metabolism, allowing the metabolites in this pathway to be consumed.

### 2.3 HCP is absorbed rapidly and scavenges multiple cyanide ions

**Figure 3:**
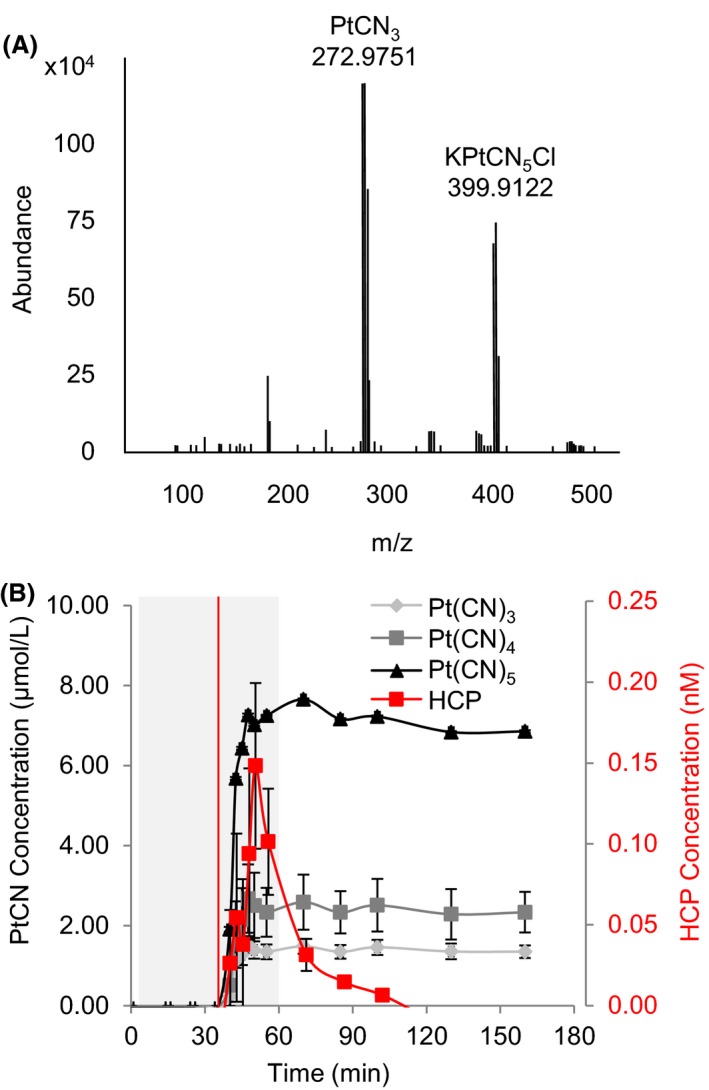
Intramuscular delivery of HCP is rapidly absorbed and scavenges multiple cyanide ions. A, Representative mass spectrograph of antidote‐cyanide profile in rabbit sera. B, Pharmacokinetic profile of HCP and HCP‐cyanide species in the sera of rabbits (n = 9). Red line denotes when antidote was delivered

Beyond improved survival and functional restoration of metabolism, a fundamental requirement for any rapidly deployable cyanide antidote is robust bioavailability via intramuscular injection. Platinum complexes contain a positively charged metal core that binds the cyanide anion, thereby mitigating the toxic effects of this poison. Using tandem mass spectrometry and isotope distribution comparison, we previously determined that cisplatin binds 3‐4 cyanide anions in an in vitro reaction.^13^ To extend prior studies, we performed pharmacokinetic analyses to monitor the associative substitution reaction of cyanide and HCP in vivo. Baseline and serial blood sampling were performed over 220 minutes and analyzed using mass spectrometry. A representative mass spectrograph is displayed in Figure 3A, demonstrating the profile of HCP‐cyanide species identified in rabbits.

Shortly after intramuscular injection of HCP at t = 40, HCP is detected in blood (t = 42.5). The observed HCP sera C_max_ was 148 ± 52 nmol/L HCP and the observed t_max_ was 7.5 minutes (Figure 3B, red line denotes antidote injection). As HCP levels diminished (half‐life = 19 minutes), we observed increased levels of several PtCN species including tricyanoplatinate, tetracyanoplatinate, and pentacyanoplatinate (Figure 3B, gray and black lines, n = 7). Sera C_max_ was 1.52 ± 0.02, 2.67 ± 0.85, and 7.65 ± 0.02 µmol/L for PtCN_3_, PtCN_4_ and PtCN_5_, respectively. Sera t_max_ was 7.5 minutes for all three HCP‐CN species. Notably, HCP‐CN species were detected 2.5 minutes post antidote injection, indicative of the rapid uptake of HCP and sequestration of cyanide by HCP. Further, HCP binds 3‐5 cyanide anions in vivo. The predominant species is PtCN_5_ displaying 5‐fold and 3‐fold greater levels than PtCN_3_ and PtCN_4_, respectively. The pharmacokinetic profiles of HCP and HCP‐cyanide species demonstrate the early bioavailability of HCP and its rapid scavenging of cyanide.

### 2.4 Surrogate biomarkers of adverse drug reactions do not change during acute HCP exposure in rabbits

**Figure 4:**
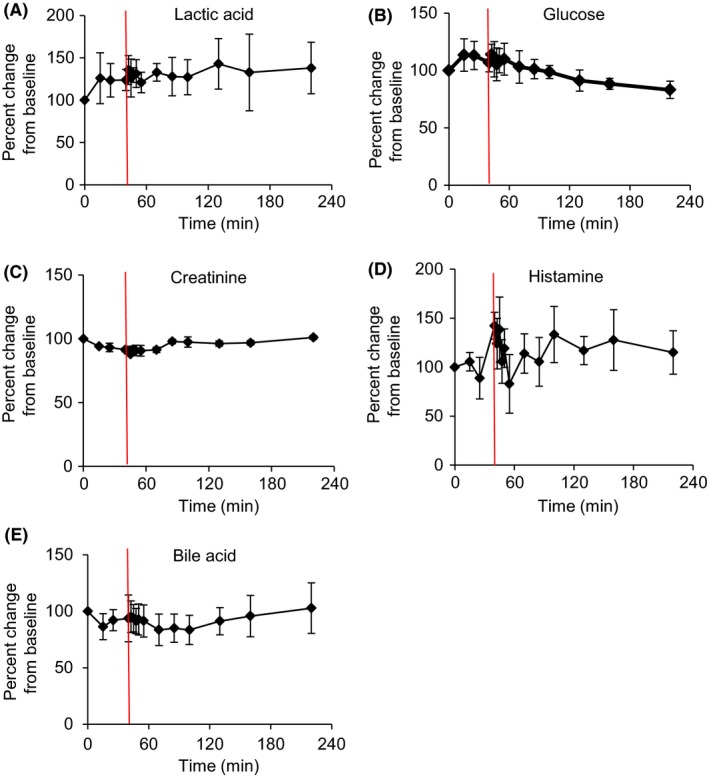
Surrogate biomarkers of adverse drug reactions do not change during acute HCP exposure in rabbits. Levels of A, lactic acid, B, glucose, C, creatinine, D, histamine, and E, bile acids in rabbits treated with HCP for 220 minutes. Red line denotes when HCP was delivered. Data were normalized to baseline and presented as mean ± SEM

Given the beneficial effects of HCP on cytochrome c oxidase function, TCA cycle metabolism, and survival, we next sought to rule out potential acute adverse drug reactions induced by administration of antidote alone. Our metabolite platform monitors surrogate markers of adverse drug reactions including creatinine (kidney function), glucose (glycemic homeostasis), histamine (allergic reaction), lactic acid (acidosis), and bile acids (liver function). Rabbits were anesthetized, ventilated and lines were placed, using the exact procedure used for cyanide infusion experiments. However, in these experiments rabbits were infused with saline for 60 minutes (n = 3). Following saline administration, HCP (30 mg/kg) was given intramuscularly, and plasma metabolite measurements were measured serially for 220 minutes (Figure 4A‐E). No significant changes were observed between baseline and t = 220 minutes across the range of metabolite biomarkers of drug toxicity excluding the major mechanisms of an acute adverse drug reaction during this timeframe. Given these findings, we next formally assessed safety in mice.

### 2.5 Toxicity studies in mice treated with HCP reveal normal blood chemistry and no major organ toxicity

Mice were treated with a single dose of vehicle (DMSO‐saline) or HCP (200 mg/kg, IM) and monitored for 4 days. Subsequently, blood was collected for Comprehensive Metabolite and Complete Blood Count panels, and all the organs were harvested for histological analysis (Table 1, 2, n = 11 HCP, n = 4 controls). This dose was selected based on our previous studies demonstrating HCP has an EC_50_ of 50 mg/kg in mice exposed to a lethal dose of cyanide.^13^

**Table 1:** Comprehensive metabolic panel results in mice 4 days after treatment with 200 mg/kg IM HCP or vehicle

Analyte	Ref. range	Control	HCP	*P* value
GLU	172‐258 mg/dL	196.00 ± 15.04	186.00 ± 10.5	0.777
BUN	16‐21 mg/dL	16.80 ± 1.25	13.70 ± 0.45	0.009
CREA	0.10 mg/dL	0.20 ± 0.00	0.20 ± 0.01	0.249
PHOS	8.6‐10.2 mg/dL	8.20 ± 0.55	9.70 ± 0.39	0.124
CA	9.5‐10.3 mg/dL	10.50 ± 0.06	10.50 ± 0.11	0.866
NA	154.4‐158.8 mmol/L	145.70 ± 0.33	146.30 ± 0.57	0.740
K	10.0‐7.8 mmol/L	4.60 ± 0.28	4.80 ± 0.19	0.625
CL	96.1‐134.1 mmol/L	107.30 ± 1.33	108.10 ± 0.78	0.755
TP	5.1‐5.7 g/dL	4.90 ± 0.03	4.90 ± 0.07	0.900
ALB	2.2‐2.4 g/dL	2.50 ± 0.03	2.50 ± 0.04	0.565
GLOB	2.9‐3.3 g/dL	2.40 ± 0.00	2.50 ± 0.03	0.349
A/G	0.8 (ratio)	1.00	1.00	0.104
ALT	37.6‐85.8 U/L	52.00 ± 9.17	40.80 ± 1.05	0.073
ALP	58.7‐104.7 U/L	119.70 ± 7.31	92.30 ± 7.78	0.099
TBIL	0.1‐0.3 mg/dL	0.50 ± 0.09	0.70 ± 0.08	0.416
CHOL	114.4‐163.4 mg/dL	106.70 ± 18.11	101.70 ± 4.16	0.715
AMY	1691‐3615 U/L	2384.50 ± 111.45	1948.80 ± 61.86	0.056

**Table 2:** Complete blood count panel results in mice 4 days after treatment with 200 mg/kg IM HCP or vehicle

CBC	Ref. range	Control	HCP	*P* value
TP (R)	5.1‐5.7 g/dL	18.10 ± 13.2	4.90 ± 0.09	0.221
RBC	8.4‐11.0 M/µL	7.61 ± 0.13	7.70 ± 0.20	0.839
HCT	47.2%‐55.7%	44.40 ± 0.90	43.90 ± 1.07	0.713
HGB	13.8‐16.6 g/dL	13.10 ± 0.20	12.50 ± 0.31	0.250
MCV	54.0‐57.8 fL	58.40 ± 1.32	57.20 ± 0.57	0.345
MCHC	29.4‐30.2 g/dL	29.50 ± 0.32	28.60 ± 0.40	0.193
RDW	%	13.10 ± 0.77	14.60 ± 0.25	0.064
WBC	4.8‐9.8 K/µL	4.50 ± 0.35	3.70 ± 0.33	0.174
SEG	K/µL	0.80 ± 0.15	0.80 ± 0.24	0.881
LYMPH	77‐8‐88.4 K/µL	3.10 ± 0.58	2.60 ± 0.12	0.359
MONO	0.9‐4.9 K/µL	0.27 ± 0.09	0.20 ± 0.08	0.655
EOS	1.2‐2.6 K/µL	0.40 ± 0.20	0.10 ± 0.02	0.066
RETIC	387.9‐400.8 K/µL	373.1 ± 39.68	825.3 ± 66.14	0.003

No acute kidney dysfunction was detected in HCP versus control treated mice as demonstrated by the lack of statistically significant differences in creatinine (0.20 ± 0.01 vs 0.20 ± 0.00 mg/dL, respectively) and phosphate levels (9.70 ± 0.39 vs 8.20 ± 0.55 mg/dL). Further electrolytes were normal in HCP‐treated mice versus controls (calcium 10.50 ± 0.11 vs 10.50 ± 0.06 mg/dL, sodium 146.30 ± 0.57 vs 145.70 ± 0.33 mmol/L, potassium 4.80 ± 0.19 vs 4.60 ± 0.28 mmol/L, and chloride 108.10 ± 0.78 vs 107.30 ± 1.33 mmol/L). Additionally, there were no significant differences in alanine aminotransferase (40.80 ± 1.05 vs 52.00 ± 9.17 IU/L), alkaline phosphatase (92.30 ± 7.78 vs 119.70 ± 7.31 IU/L), and total bilirubin (0.70 ± 0.08 vs 0.50 ± 0.09 mg/dL) in HCP compared to control treated mice, respectively, excluding early hepatotoxicity. Amylase, a marker of pancreatic dysfunction, was not significantly different between controls (2384.50 ± 111.45 IU/L) and HCP (1948.80 ± 61.86 IU/L) treated animals. Blood urea nitrogen, BUN, (13.70 ± 0.45 vs 16.80 ± 1.25 mg/dL; *P* = 0.009) was statistically different between groups, but both groups were lower than the normal reference range for CD‐1 mice indicative of an underlying factor affecting BUN in these particular mice (19‐29 mg/dL). Cumulatively, these clinical findings demonstrate that 200 mg/kg IM HCP is well tolerated by mice.

A Complete Blood Count panel revealed no significant differences in erythrocytes, leukocytes, eosinophils, monocytes,or lymphocytes (Table 2). The only significant difference was increased reticulocytes (825.3 ± 66.14 vs 373.1 ± 39.68 K/µL; *P* = 0.003). For reference, the normal reticulocyte values in CD‐1 mice range between 200 and 500 K/µL, which in general is higher than most other species due to their relatively short life spans.^15^ Acute muscle necrosis was identified at the injection site (hind limb) on gross and histologic evaluation (Supplementary Figure S1). Neutrophils and erythrocytes were primarily associated with the necrotic muscle, and inflammation occasionally extended into the deep subcutis. Formulation studies are currently underway to overcome HCP‐induced injection site injury. Pathologic lesions were not identified in the lung, heart, liver, spleen, kidney, and brain. In summary, these preliminary toxicity studies demonstrate that HCP is well tolerated and further the most concerning toxicity anticipated for platinum analogs, that is renal damage is not observed after HCP administration.

### 2.6 Replication of survival, pharmacokinetic, and metabolite findings in a swine model of cyanide poisoning

**Figure 5:**
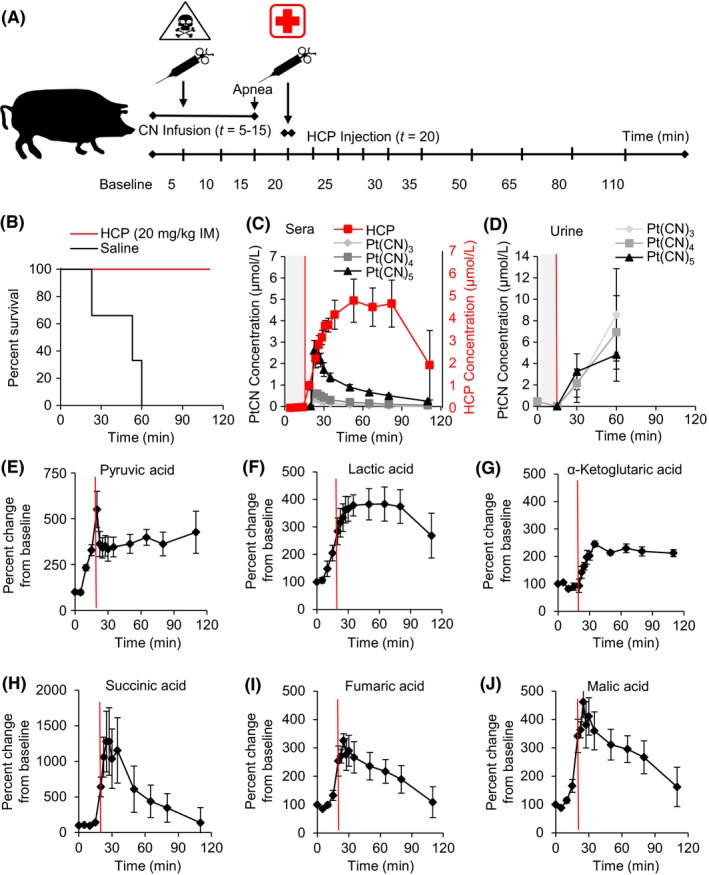
Replication of survival, pharmacokinetic, and metabolite findings in a swine model of cyanide poisoning. A, Overview of experimental procedure in the swine cyanide model. B, Kaplan‐Meier plot of HCP (red) and saline (black) treated pigs exposed to a lethal dose of cyanide. Pharmacokinetic profile of HCP and HCP‐cyanide species in the sera C, and urine D, of pigs (n = 3). Levels of E, pyruvic acid, F, lactic acid, G, α‐ketoglutaric acid, H, succinic acid, I, fumaric acid, and J, malic acid in pigs treated with cyanide (at t = 1) and given HCP (at t = 20 minutes, n = 3). Red line denotes when antidote was delivered

Motivated by the favorable pharmacokinetics, efficacy and safety of HCP in small mammals, we conducted a pilot study to test the efficacy of HCP in a swine model. Our model is designed to mimic an out of hospital, acute cyanide exposure scenario, such as during a fire or a terrorist attack.^10^ Animals are not mechanically ventilated, allowing them to become apneic following intravenous administration of potassium cyanide. Pigs are held at apnea for 5 minutes and subsequently administered with saline or antidote. Invasive blood pressure is monitored continuously and blood sampling occurs serially throughout both the exposure and recovery phases of the model. In saline‐treated control animals, death occurs in 100% of cases within 60 minutes following cyanide exposure.^16^ Whereas, pigs treated with 20 mg/kg HCP IM (n = 3) all survived to the endpoint of the study, 110 minutes after commencing cyanide infusion (Figure 5A‐B). These preliminary findings suggest HCP also confers protection in a swine model of severe, lethal cyanide poisoning.

Shortly after intramuscular administration of antidote, we detected 0.99 ± 0.18 µmol/L of HCP in the circulation at 5 minutes post antidote injection (Figure 5C), similar to the rapid absorption in the rabbit model (2.5 minutes post injection, Figure 3B). The concentration of HCP steadily rose over the next 30 minutes, reaching a C_max_ of 4.79 ± 0.95 µmol/L at 35 minutes post antidote injection. Subsequently, HCP concentration plateaued. By t = 110 (95 minutes post antidote injection), the levels declined to 1.91 ± 1.50 µmol/L, exhibiting a half‐life of 91 minutes in this model (Figure 5C). Similar to our observations in the rabbit model, HCP formed HCP‐CN_3_, ‐CN_4_ and ‐CN_5_ species in swine model. As in the rabbit model, PtCN_5_ is the predominant species. In this model, PtCN_5_ displayed a C_max_ of 2.60 ± 0.47 µmol/L and a t_max_ of 12.5 minutes (Figure 5C). In contrast to the rabbit model, HCP was cleared slower and HCP–CN species were cleared faster in the swine model.

At t = 110, no PtCN species were detectable in the blood. PtCN_5_ displayed a half‐life of 17 minutes. Therefore, we next evaluated excretion of HCP–CN species in the urine at baseline and at various time‐points post antidote delivery (Figure 5D). We detected increasing concentrations of all three PtCN species in the urine following antidote injection. The highest concentration detected in the urine was at the last time‐point measured (PtCN_3_ = 8.5 ± 4.3, PtCN_4_ = 6.9 ± 3.4, and PtCN_5_ = 4.8 ± 2.4 µmol/L, respectively, Figure 5D). These findings demonstrate that, in a large mammalian model, HCP is rapidly absorbed after intramuscular administration, quickly reacts with cyanide, binding up to five cyanide anions, and HCP–CN species are excreted into the urine.

Metabolite profiling revealed many similarities in HCP‐induced effects in the swine compared to the rabbit, though some differences were observed (Figure 5E‐J). Concordant with the rabbit model, the most pronounced changes were in succinic acid (+1277% ± 426%; *P* = 0.05, Figure 5H); significant increases in α‐ketoglutaric acid (+245% ± 10%, *P* = 0.03), fumaric acid (+325% ± 23%, *P* = 0.04), and malic acid (+462% ± 54%, *P* = 0.001) were also observed. In rabbits, TCA cycle metabolites accumulate during the cyanide infusion period and slowly return to toward baseline levels (Figure 2). Even more pronounced relief of TCA cycle block was observed in the pig model following HCP injection: succinic acid (+137% ± 213%, Figure 5H), fumaric acid (+108% ± 54%, Figure 5I), and malic acid (+162% ± 69%, Figure 5J). Malic acid peaked at +245% ± 66% and remained elevated (+212% ± 62%) in contrast to the rabbit model in which this metabolite trended towards baseline (Figure 2F).

Interestingly, injection of HCP had immediate effects on pyruvate metabolism in the swine model that was not observed in the rabbit model (Figure 2A). In the swine model, infusion of cyanide resulted in peak pyruvate levels at t = 20 minutes (+550% ± 98%; *P* = 0.001, Figure 5E). At this point antidote was injected and after 2.5 minutes post antidote administration, we observed peak levels of PtCN_5_. Over the same period of time there was a significant drop in pyruvate levels (+550% ± 98% to +362% ± 68%; *P* = 0.05). During the remainder of the experimental protocol, pyruvate levels remained steady. Lactic acid levels increased to +362% ± 48%, plateaued and began to decline at t = 90 (Figure 5F), whereas in the rabbit model lactic acid plateaued and remained elevated (Figure 2B). Compared to the rabbit model, these data demonstrate concordant effects on TCA cycle metabolism, in addition to unique metabolic effects in the pig model. Together the findings in the swine study demonstrate that HCP was rapidly absorbed by intramuscular administration, activated TCA cycle metabolism, and improved survival.

## 3 DISCUSSION

Here we demonstrate that intramuscular administration of HCP is an effective antidote to a lethal dose of cyanide in rabbits. In a pilot study in a second mammalian model, we provide proof‐of‐concept data that HCP also improves survival in pigs. We determined the pharmacokinetic profiles in rabbits and pigs. In both models, HCP is rapidly absorbed and detected in the systemic circulation minutes post‐IM injection. Concordantly, we observe rapid binding of HCP to cyanide, the predominant reaction product being PtCN_5_ in both models. Further, HCP restores cellular physiology as assessed by the functional parameters of cytochrome c oxidase redox state and oxy‐/deoxy‐hemoglobin. Finally, HCP alleviated the metabolic derangements induced by cyanide and activated TCA cycle metabolism.

Currently used FDA‐approved antidotes to cyanide include sodium thiosulfate/amyl nitrite and hydroxocobalamin. Hydroxocobalamin acts by directly binding and sequestering cyanide; its stoichiometry only allows for equimolar amounts of cyanide to be bound by the drug. The treatment is limited by its low solubility in water, therefore necessitating large volumes of drug administration to counteract cyanide toxicity. Here, we demonstrate that HCP binds up to five cyanide anions, improving the stoichiometry for binding multiple cyanide anions when compared to existing chelators or other antidotes. Additionally, dissociation of cyanide from the hydroxocobalamin‐cyanide complex prior to excretion of the complex is a potential concern, necessitating that patients be monitored for several days after hydroxocobalamin treatment. By contrast, the bond between platinum and cyanide has very high affinity at physiological pH.^17^ Further, we demonstrated that PtCN complexes do not degrade and release cyanide that subsequently induces secondary toxicity.^13^ Therefore, the chemical and pharmacokinetic properties described in these preclinical studies of HCP suggest several potential advantages over current FDA‐approved antidotes.

In addition to evaluating if HCP is an effective antidote by intramuscular delivery, we sought to determine the in vivo pharmacokinetic profile in rabbits and pigs. The concordant detection of HCP and PtCN species in the sera of both mammalian models suggests rapid and robust sequestration of cyanide by HCP. However, there are several notable differences in the pharmacokinetic profile in the swine model compared to the rabbit model. The half‐life of HCP is significantly longer in the swine model compared with the rabbit model (91 vs 19 minutes). This may be due to differences in drug metabolism between the species. Of note, compared to other experimental animals, porcine enzymes more closely resemble the activity of human cytochrome P450 and other enzymes responsible for xenobiotic biotransformations, and therefore are regarded as a favored model for ADME studies.^18^, ^19^ In both species, PtCN_5_ is the predominant cyanoplatinum species formed. However, in rabbits, HCP‐cyanide species peak and plateau, remaining elevated for the remainder of the experiment. By contrast, in pigs, HCP‐cyanide species peak, steadily declines, and are undetectable by the end of the experiment (half‐life of PtCN_5_ = 17 minutes). Concomitantly, as the levels of PtCN species begin to decline in the sera, they become detectable in the urine. Thus, PtCN species are rapidly formed and cleared from the circulation via renal excretion. We acknowledge that the swine study is a pilot study and must be replicated in a larger cohort, none‐the‐less; these preliminary results are important because swine models closely resemble humans in physiology. Taken together, our pharmacokinetic findings in the rabbit and swine models provide strong evidence that HCP is a favorable lead compound for the development of an intramuscular deployable countermeasure to cyanide poisoning.

In addition to displaying a favorable pharmacokinetic profile, HCP also activated the TCA cycle metabolism as demonstrated by the rapid consumption of TCA cycle metabolites following antidote administration. The most pronounced TCA metabolite changes during cyanide infusion were in succinic acid (+1907% in rabbits and +1277% in pigs). The levels were significantly abrogated following HCP administration indicating that HCP activates TCA cycle metabolism, allowing the metabolites in this pathway to be consumed. Whereas the surrogate cyanide biomarker lactate increased in rabbits and pigs +429 and +362, respectively. Interestingly, lactate levels plateaued but did not return to baseline. From the lactate measurements that we have collected over the years, we typically detect a rise in lactate levels during cyanide poisoning, as expected, and the levels continue to rise until death ensues. Delivery of an efficacious antidote often results in lactate levels falling. However, we also observe instances in which efficacious antidotes result in lactate levels plateauing, whereas in the absence of antidote the levels continue to rise.^16^ This may be due to the relatively short duration of the study. Concomitantly, in the last time‐point collected in the swine study (90 minutes post antidote injection), the lactate levels begin to decline (Figure 5F). However, these findings also highlight an important knowledge gap in our understanding of cyanide toxicity. Although cyanide has long been described as a major disruptor of metabolic homeostasis, metabolomics profiling studies in mammalian models of cyanide poisoning are lacking.^20^ In the case of the data presented here, TCA cycle metabolites return to near baseline levels suggesting reactivation of the TCA cycle but, curiously, both pyruvate and lactate remain elevated. Further metabolic profiling studies across a range of efficacious antidotes are required to fully understand the breath of metabolic changes that occur during the prevention of cyanide poisoning. Therefore, we plan to characterize the metabolic profile observed after treatment with a range of experimental cyanide antidotes being studied by the community.

HCP emerged from our previous structure‐activity relationship studies of cisplatin analogues. In early development of this compound, we observed that ligands complexed to the platinum core greatly affected efficacy as a cyanide countermeasure.^13^ Of the many complexes evaluated, HCP exhibited substantially improved solubility and reduced cytotoxicity in human cells, zebrafish, mice, and rabbits.^13^ Toxicity is a concern with all platinum‐based drugs. However, cisplatin has been used clinically for over 50 years and is first‐line treatment for many types of solid tumors despite its known nephrotoxicity, neurotoxicity, and ototoxicity.^21^, ^22^, ^23^, ^24^ While acute cytotoxic effects of cisplatin do occur, most of side effects arise from repeated cycles and long‐term administration. Encouragingly, HCP's reported toxicity in mice is ~20‐fold lower than cisplatin (LD50 = 133 vs 6.6 mg/kg, intraperitoneally).^14^, ^25^ In contrast with cisplatin, few mammal toxicity studies have been conducted to systematically analyze HCP. A rabbit study of subcutaneous injection of HCP determined that the LD_Lo_ was 180 mg/kg; this dose is six times higher than the dose used to rescue rabbits from a lethal dose of cyanide.^25^ Consistent with these findings, we found that 30 mg/kg IM HCP in rabbits did not alter metabolite biomarkers of drug toxicity. Further, here we demonstrate that HCP treatment in mice did not induce major organ toxicity as demonstrated by blood chemistry panels and organ histology 4 days following the administration of 200 mg/kg HCP IM. The only lesion observed was muscle necrosis at the injection site. Notably, our preliminary toxicity studies demonstrate that the most concerning toxicity anticipated for platinum analogs that is renal damage was not observed after HCP administration.

In the future, formulation studies will be required to mitigate the injection site injury. Formal preclinical toxicology experiments are beyond the scope of this study but will also be required to fully characterize HCP. An additional area of future investigation is the capacity of HCP to mitigate cyanide toxicity from nitroprusside administration. Cumulatively, these findings establish HCP as a promising lead compound that (a) demonstrates substantial bioavailability binding cyanide minutes post‐IM injection with subsequent urinary excretion of the complex; (b) enables reactivation of metabolism; (c) restores parameters of cellular physiology; (d) improves survival in two mammalian models; and (e) exhibits minimal acute toxicity. The favorable efficacy, pharmacokinetic and safety profiles of the HCP in mice, rabbits, and pigs warrant further development of HCP as a rapidly deployable cyanide antidote.

## 4 METHODS

### 4.1 Preparation of HCP

To 120 mg sodium HCP, we added 120 µL of DMSO. The tube was placed in a 95°C water bath for a few minutes and vortexed for 2 minutes. Then 1.26 mL of the Ca^2+^, Mg^2+^‐free PBS solution (at 95°C) was added to the tube and further vortexed for 2 minutes. This solution was delivered intramuscularly.

### 4.2 Rabbits cyanide model

Pathogen‐free male and female New Zealand White rabbits (Western Oregon Rabbit Supply, Philomath, OR) weighing 3.5‐4.5 kg were used. All animals were anesthetized with ketamine and xylazine, intubated, and ventilated at a respiratory rate of 20‐22 breaths/min, a tidal volume of 60 cc, and fraction of inspired oxygen (FiO_2_) of 100%. Arterial and venous blood samples were drawn at the indicated time points. Rabbits were monitored for cyanide poisoning in real time using standard hemodynamics, gas exchange measures, and optical technologies including continuous wave near infrared spectroscopy DOS as previously described.^26^, ^27^ A lethal cyanide dose was achieved by intravenous administration of 22‐26 mg sodium cyanide in 60 mL of saline at 1 cc/min (0.33 mg/min). The 100% O_2_ supply was switched to atmospheric air after 30 minutes of CN infusion and the respiratory rate on the ventilator was reduced down to 18‐20 breaths/min. When the blood pressure dropped below 40‐58 mmHg, antidote or placebo (saline) was administered (IM into the right front limb muscle) and cyanide was continued for another 30 minutes. This results in 80% lethality unless the antidote is effective. Animals that survive are monitored for an additional 160 minutes. For this study, saline rather than vehicle was used in the control animals as we previously used the vehicle DMSO‐saline formulation compared to HCP in a lethal model of cyanide poisoning and found that the vehicle formulation does not affect survival.^13^ All the methods were carried out in accordance with the regulations and guidelines of the Animal Welfare Act and the American Association for Accreditation of Laboratory Animal Care. All experimental protocols were approved by the IACUC committee at UC Irvine.

### 4.3 Swine cyanide model

Female Yorkshire swine (*Sus scrofa)* (Oak Hill Genetics, Ewing, IL) weighing 45‐55 kg were used for this study. We selected females due to ease of urinary catheterization. The sigmoid flexure of the penis makes it difficult/impossible to catheterize male swine.^28^ Anesthesia is induced with intramuscular administration of 10‐20 mg/kg ketamine and isoflurane (MWI, Boise, ID) via nosecone. Following induction, animals are intubated with an 8.0 cuffed endotracheal tube (Teleflex, Morrisville, NC), and peripheral venous access obtained. Sedation is maintained using the Drager Fabius GS anesthesia machine (Drager, Houston, TX) with 1%‐3% isoflurane and 0.4 FiO_2._ Tidal volume is set at 8 ml/kg and a respiratory rate 16‐20 breaths/min, adjusting the minute volume to maintain an end tidal CO_2_ of 35‐45 mmHg. A 7.5 mL/kg bolus of 0.9% saline (B. Braun, Bethlehem, PA) is given prior to central line placement. The external jugular and femoral artery are visualized using the M9 ultrasound system (Mindray, Mahwah, NJ) and central venous and arterial access obtained. The Drager Infinity Delta Monitor (Drager, Houston, TX) monitors and records pulse oximetry, body temperature, invasive blood pressure, and ECG throughout the experiment. Invasive hemodynamic variables are measured via pulmonary artery catheterization using an eight‐French Swan Ganz CCOmbo catheter and the Edwards Vigilance II monitor (Edwards Lifesciences, Irvine, CA). Once vascular access is obtained, a one‐time bolus of heparin (100 units/kg) is administered intravenously. Mechanical ventilation is then terminated, allowing the animal to breathe spontaneously and isoflurane as well as FiO_2_ are weaned to 0.8%‐1% and 0.21, respectively.

The control animals were treated with intramuscular saline in place of the antidote. These controls are run periodically throughout the year and we observe negligible cohort to cohort variation. Potassium cyanide (Sigma Aldrich, St. Louis, MO) diluted in saline (B. Braun, Bethlehem, PA) is delivered via continuous infusion into the right jugular vein until 5 minutes after apnea occurs. At this point, animals are treated with either HCP or saline injected into the left gluteal muscle and the cyanide infusion is terminated. Following the treatment, animals are observed continuously for 90 minutes or until death, defined as a mean arterial pressure of less than 20 for 10 minutes. At the end of the study, all animals are euthanized with an intravenous administration of 100 mg/kg sodium pentobarbital. All methods were carried out in accordance with the regulations and guidelines of the Animal Welfare Act and the American Association for Accreditation of Laboratory Animal Care. All experimental protocols were approved by the IACUC committee at the University of Colorado.

### 4.4 Pharmacokinetics of PTCN and PTCL species

To generate standard curves for the observed chloroplatinate and cyanoplatinate species, K_2_PtCl_4_, Na_2_PtCl_6_•6H_2_O, and K_2_Pt(CN)_4_ standards (Sigma Aldrich) were dissolved in normal saline to generate 10 mmol/L stock solutions. A hexachloroplatinate‐DMSO (HCP‐DMSO) solution (10 mmol/L) was prepared by adding 5.6 mg of Na_2_PtCl_6_•6H_2_O into 1 mL of 95°C DMSO followed by incubation in the dark for 1 hour. A solution of 1 mol/L potassium cyanide (pH 7.4 adjusted with sodium hydroxide) was prepared and then equal volumes of the HCP‐DMSO and potassium cyanide solutions were combined, vortexed, and incubated in order to generate the cyanoplatinum complexes. Calibration standards with concentrations ranging from 0.00095 μmol/L to 1000 μmol/L were generated by spiking 2 μL of 10 mmol/L solution into 18 μL of pooled plasma followed by serial dilutions in pooled plasma. The prepared calibration samples (10 μL) were deproteinized using 90 μL of 75:25 methanol:acetonitrile with isotopically labeled internal standards (phenylalanine d8 and valine d8). Samples were vortexed for 5 seconds and then subjected to centrifugation (14 000*g*, 20 minutes, and 4°C). Supernatants were transferred to glass autosampler vials with 300 μL inserts for analysis. Experimental samples were aliquoted (10 μL) and prepared using the same sample preparation workflow.

LC‐MS data were acquired using a Hilic Chromatography on a 2.1 × 150 × 3.5 μm Atlantis HILIC column (Waters, Milford, MA). The chromatography system was an Agilent 1200 series LC with a CTC PAL Autosampler. Mobile phase A consisted of 10 mmol/L ammonium formate in water with 0.1% formic acid, and mobile phase B consisted of 100% acetonitrile, with 0.1% formic acid (all components were Optima LC‐MS grade, Fisher Scientific, Hampton, NH). The injection volume was 10 μL. Initial mobile phase conditions were 5% mobile phase A, 95% mobile phase B followed by a constant gradient to 60% mobile phase A, 40% mobile phase B over 10 minutes. The column was then re‐equilibrated to initial mobile phase conditions over 20 minutes. The chromatography system was coupled to an Applied Biosciences/Sciex 4000 QTRAP mass spectrometer with an electrospray ionization source run in negative mode. MRM transitions were optimized for each species on the LC‐MS system using Analyst Software (Sciex, Framingham, MA). Following optimization, calibration standards were run to generate a standard curve for each species. Next, experimental samples were run and signals for each PtCl and PtCN species were compared to the calibration curve in order to determine their absolute concentration.

### 4.5 Targeted metabolomics

Metabolites were measured in rabbit and pig serum using adapted LC‐MS methods previously developed by our group.^29^ In this method, 30 μL aliquots were deproteinized using a 75:25 methanol:acetonitrile solution with isotopically labeled internal standards (citrulline D7, 10 μmol/L, inosine ^15^N_4_, 25 μmol/L, phenylalanine D8, 10 μmol/L, and thymine d4 25 μmol/L). Following vortexing (5 seconds) and centrifugation (14 000*g*, 20 minutes, 4°C), the supernatants were transferred to glass autosampler vials with 300 μL inserts for analysis.

LC‐MS data were acquired using HILIC chromatography on a 2.1 × 100 mm × 3.5 μm XBridge Amide column (Waters, Milford, MA) in negative ion mode. The chromatography system was an Agilent 1290 infinity HPLC coupled to an Agilent 6490 triple quadrupole mass spectrometer with an electrospray ionization source. Mobile phase A was 95:5 (v/v) water:acetonitrile (Fisher Scientific, Hampton, NH) with 20 mmol/L ammonium acetate (Sigma Aldrich, St. Louis, MO) and 20 mmol/L ammonium hydroxide (Sigma Aldrich, St. Louis, MO) (pH 9.5). Mobile phase B was 100% acetonitrile (Fischer Scientific, Hampton, NH). Injection volume was 5 μL. The mass spectrometry settings were: sheath gas temperature 400°C, sheath gas flow 12 L/min, drying gas temperature 290°C, drying gas flow 15 L/min, capillary voltage 4000 V, nozzle pressure 30 psi, nozzle voltage 500 V, and delta EMV 200 V.

LC‐MS data were quantified using Agilent MassHunter Quantitative Analysis software. All metabolite peaks were manually reviewed for peak quality in a blinded manner. Pooled plasma was interspersed throughout the run at regular intervals in order to monitor the temporal drift in mass spectrometry performance. Metabolites were normalized to the baseline sample acquired for each animal on a metabolite‐by‐metabolite basis.

### 4.6 Murine toxicity study

Male (n = 8) and female (n = 8) CD‐1 mice (Envigo, Indianapolis, IN) aged 3‐4 weeks with weight ranges of 18‐20 g were used for this study. Studies were performed at the Purdue Translational Pharmacology and Clinical Veterinary Pathology Laboratories which have IACUC approval to conduct toxicity studies. HCP was administered by intramuscular injection in 50 µL of HCP solution (10% DMSO in Ca^2+^ and Mg^2+^‐free PBS) into the gastrocnemius muscle. Control animals received 50 µL of the vehicle (10% DMSO in Ca^2+^ and Mg^2+^‐free PBS) to avoid any confounding variability due to formulation. Blood was drawn 4 days post HCP injection (200 mg/kg IM) for conducting a Comprehensive Metabolite Panel and Complete Blood Count Panel. At the end of the study, the animals were humanely euthanized following the PHS Policy on the Human Care and Use of Animals, Guide for the Use and Care of Laboratory Animals. All major organs were harvested and subjected to histological analysis by a trained pathologist. All methods were carried out in accordance with the regulations and guidelines of the Animal Welfare Act and the American Association for Accreditation of Laboratory Animal Care. All experimental protocols were approved by the IACUC committee at Purdue University.

### 4.7 Statistics

For the rabbit and pig data, significance was assessed using paired *t* tests to compare the peak areas of baseline (precyanide infusion) samples to end of the cyanide infusion samples. In both models, metabolite differences were then compared in baseline samples vs endpoint samples in order to demonstrate amelioration and a trend toward normalization by the experimental endpoint. To determine the percent change from baseline, we normalized metabolite levels in animals treated with cyanide to the value of their baseline (precyanide infusion) peak area on a metabolite by metabolite basis, and then calculated the mean and standard error of the mean. In mice, the results of the Comprehensive Metabolite and Complete Blood Count Panels were compared between mice treated with HCP and mice treated with normal saline using Student's *t* tests. Males and females were also compared separately to determine if gender specific changes had occurred.

## 5 CONFLICT OF INTEREST

The authors declare no competing interests.

## AUTHOR CONTRIBUTIONS

J.M., V.S.B., S.M., M.B., and A.K.N. designed the study; J.M., J.L., T.H.H., and A.W. carried out experimental investigation; T.L., G.K., and V.J.D. conducted Murine Toxicity Studies; J.M., J.L., T.H.H., A.W., T.L., G.K., C.A.M., G.R.B., R.T.P., V.J.D., R.E.G., V.S.B., S.M., M.B., and A.K.N. performed data analysis, reviewed and edited the manuscript; J.M. and A.K.N. written the original draft; R.E.G., M.B., S.M., V.S.B., and A.K.N. performed supervision.
