# Supplementary material for: Intramuscular administration of hexachloroplatinate reverses cyanide‐induced metabolic derangements and counteracts severe cyanide poisoning
Source: FASEB Bioadv. 2018 Nov 15;1(2):81–92. doi: 10.1096/fba.1024 (PMC6660183; doi:10.1096/fba.1024)
Supplement: Supplementary file 1 [file FBA2-1-81-s001.pdf]

Supplemental Figure 1. Acute myonecrosis at the injection site.

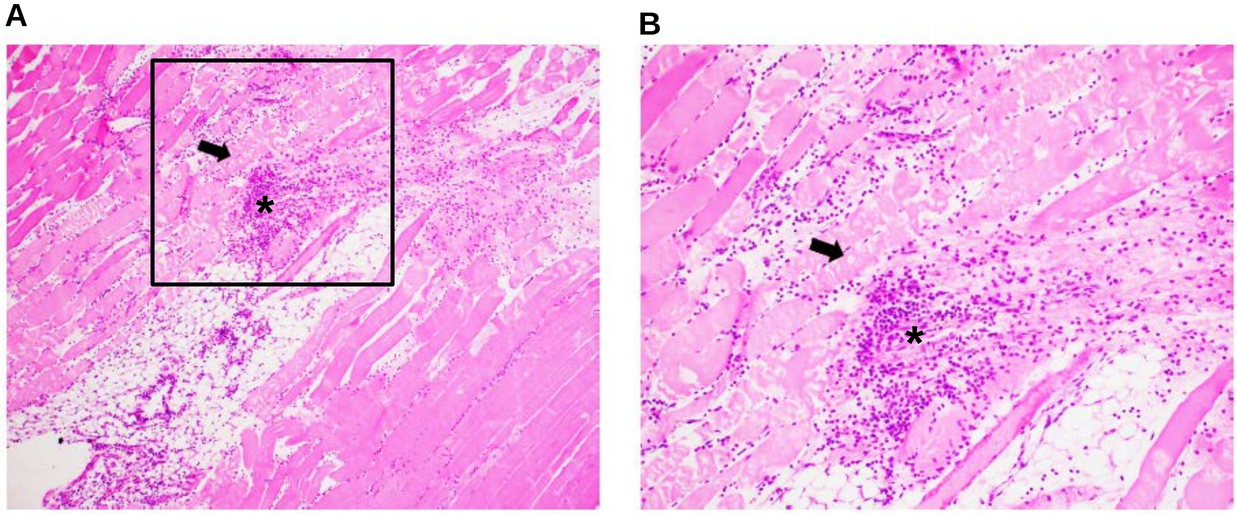

A) Multifocal myocytes (black arrow) were hyper-eosinophilic, fragmented with pykonsis, and lacked cross striations. Multifocal, necrotic myocytes were infiltrated by neutrophils (asterisk). These findings are consistent with necrosis at the injection site. **B)** Higher magnification of region denoted by the black box.
